# Persistent Adult-Onset Attention-Deficit/Hyperactivity Disorder (ADHD) Manifesting as Occupational Impairment: Highlighting the Therapeutic Potency of Methylphenidate

**DOI:** 10.1192/j.eurpsy.2024.998

**Published:** 2024-08-27

**Authors:** P. S. Gopan

**Affiliations:** Psychiatry, Surabhi medical college, Siddipet, India

## Abstract

**Introduction:**

This case study emphasizes the significance of considering unrecognized adult-onset ADHD, particularly in patients with chronic forgetfulness and occupational inefficiencies refractory to standard treatment options. The case outlined involves a 33-year-old male with enduring cognitive impairments, leading to Extreme Anxiety Disorder with detrimental consequences on his professional progression and personal well-being.

**Objectives:**

This necessitates the need for advanced research initiatives and broader awareness programs to facilitate improved diagnostic accuracy and optimization of therapeutic outcomes. Emphasizing ADHD as a potential cause of such symptomatology in adults and integrating effective treatment options can potentially pave the way to personalized therapeutic protocols.

**Methods:**

The patient was approached via meticulous reconsideration of previous unsuccessful treatment paradigms that primarily included antidepressants and anxiolytics, which yielded cyclical patterns of negligible amelioration, compounded by intermittent emergence of suicidal ideation. Given the limited response, a differential diagnosis of Adult-Onset ADHD was entertained.

**Results:**

The therapeutic intervention involving Methylphenidate administration led to a remarkable enhancement in the patient’s mental health and occupational efficiency. Progress was also evidenced in the patient’s improved confidence and self-esteem, with critical implications for his professional and personal life dynamics.

**Conclusions:**

This case study underscores the transformative potential of precise ADHD management in adults with chronic cognitive impairments. Further research studies involving larger cohorts are warranted to enhance the understanding of adult ADHD, its prevalence, and therapeutic strategies, which could serve as key elements in improving the overall quality of life for these patients.

**Disclosure of Interest:**

None Declared

